# Breast Cancer: A Global Concern, Diagnostic and Therapeutic Perspectives, Mechanistic Targets in Drug Development

**DOI:** 10.34172/apb.2021.068

**Published:** 2020-10-14

**Authors:** Gul-e-Saba Chaudhry, Rehmat Jan, Abdah Akim, Muhammad Naveed Zafar, Yeong Yik Sung, Tengku Sifzizul Tengku Muhammad

**Affiliations:** ^1^Institute of Marine Biotechnology, University Malaysia Terengganu, 21030 Kuala Terengganu, Malaysia.; ^2^Department of Environmental Sciences, Fatima Jinnah University, Rawalpindi, Pakistan.; ^3^Department of Biomedical Sciences, Universiti Putra Malaysia, Seri Kembangan, Selangor, Malaysia.; ^4^Department of Chemistry, Quaid-i-Azam University, Islamabad, 45320, Pakistan.

**Keywords:** Apoptosis, Breast cancer, Cell cycle arrest, Drug development, Natural products, Mechanism of action, Phytochemicals, Plant anticancer drugs

## Abstract

Cancer is a complex multifactorial process, unchecked and abrupt division, and cell growth—conventional chemotherapy, along with radiotherapy, is used to treat breast cancer. Due to reduce efficacy and less survival rate, there is a particular need for the discovery of new active anticancer agents. Natural resources such as terrestrial/marine plants or organisms are a promising source for the generation of new therapeutics with improving efficacy. The screening of natural plant extracts and fractions, isolations of phytochemicals, and mechanistic study of those potential compounds play a remarkable role in the development of new therapeutic drugs with increased efficacy. Cancer is a multistage disease with complex signaling cascades. The initial study of screening whole extracts or fractions and later the isolation of secondary compounds and their mechanism of action study gives a clue of potential therapeutic agents for future drug development. The phytochemicals present in extracts/fractions produce remarkable effects due to synergistically targeting multiple signals. In this review, the molecular targets of extracts/ fractions and isolated compounds highlighted. The therapeutic agent's mechanistic targets in drug development focused involves; i) Induction of Apoptosis, ii) modulating cell cycle arrest, iii) Inhibition or suppression of invasion and metastasis and iv) various other pro-survival signaling pathways. The phytochemicals and their modified analogs identified as future potential candidates for anticancer chemotherapy.

## Introduction

Breast cancer is the second leading cause of death (11.6% of the total cancer deaths), followed by colorectal and lung cancer.^1^ Cancer is a complex disease concerning pathology and biochemistry. It begins when cells in the body start to grow in an uncontrolled and abnormal manner, which may also cause disturbances and alter the structure of surrounding tissues.^2^ The evasion of apoptosis, limitless replicative potential, evading growth suppressors, sustaining proliferative signaling, inducing angiogenesis and activating tissue invasion and metastasis are critical features of cancer which contribute towards tumor development.^3,4^ Alterations in cellular DNA and transcriptional/translational processes causes irregularity in the gene expressions and results in cancer cell proliferation. Primary entities involved in carcinogenesis are oncogenes and tumor suppressor genes. Defects in tumor suppressor genes and mutations in the proto-oncogenes results in uncontrolled multiplication of cells leading to cancer.^5,6^ Breast cancer, lung cancer, and colorectal cancer are frequently occurring cancer in both men and women.^7^

## Breast cancer: a global concern

Breast cancer triggers due to the uncontrolled multiplication of cells. It is the most frequently occurring cancer type and the leading cause of death in women over the last few years.^8^ The cancer trigger due to mutations in genes responsible for the production of pro-apoptotic/anti-apoptotic proteins, tumor suppressers proteins, and growth factors. According to the United States, cancer statistics report 2018, an estimate of about 268 670 new breast cancer cases and a total of 41 400 deaths cases due to breast cancer in the United States in 2018.^9^ Breast cancer broadly categorized into two types, invasive breast cancer, and non-invasive breast cancer. However, other types of breast cancer include medullary and tubular carcinoma, inflammatory breast cancer,Paget’s disease (PD), and phyllodes tumor (PT).Generally, in invasive breast cancer, cells are not only confined to ducts and lobular walls but also spread to surrounding areas of breast (connective and fatty tissues). The infiltrating-lobular-carcinoma and infiltrating-ductal-carcinoma are frequently occurring invasive-breast cancer. The lobular carcinoma isinitiated in the milk glands, while ductal carcinoma began in the breast’s milk ducts. Medullary breast carcinoma and tubular carcinomas are the subtypes of invasive breast carcinoma.^10^ Furthermore, inflammatory breast cancer type is characterized by inflamed breasts with indentation and thick ridges. Only 1% to 2% of all invasive breast cancers and 1% of all breast cancers are inflammatory breast cancer with low survival rates at all stages. However, non-invasive breast cancer cells restricted to ducts only (do not penetrate surrounding tissues) of the breast. Ductal carcinoma in situ and lobular carcinoma in situ are the two forms of non-invasive breast cancer.^10-13^ PD pharmaceutically described by the infiltration of the nipple epidermis by destructive breast epithelial cells. PD of the breast defined as a skin alteration in the nipple-areola region. It is less common and generally linked with *in-situ* or invasive carcinoma.^14^ Breast PT, is a rare tumor, and shows different behavior, as it could be benign (non-cancerous) or malignant (cancerous).^15^ PT can cause uncommon fibroepithelial lesions to account for around 0.3% to 0.5% of breast tumors diagnosed in women and has an occurrence of about 2.1 per million.^16^

Breast cancer usually classified as two types i) estrogen receptor-positive (ER+) and ii) estrogen receptor-negative (ER-) breast cancer. Estrogen receptor-positive cell lines include MCF-7 and T-47D, while MDA-MB-231, MDA- MB-453 and MDA-MB-468 are estrogen negative receptor cell lines. Which, further characterized as luminal A (ER+, PR+, HER2-), luminal B (ER+, PR+, HER2+), HER2-enriched, basal-like, and normal-like based on progesterone receptor (PR) and human epidermal growth factor receptor-2 (HER2) status.^17^ The MCF-7 and T-47D cell line together with other breast cancer cell type MDA-MB-231, have been studied in above two-third of the total publications in Medline.^18^

## Risk factors, diagnostic and therapeutic perspective for breast cancer

Common risk factors associated, such as age, gender, family history, breast density, radiation exposure, reproductive factors, genetic mutations, and diabetes.^19^ Early screening, detection, and diagnosis, significantly affect the occurrence and survival rate of breast cancer.Several diagnostic approaches include mammograms, ultrasound, magnetic resonance imaging, breast self-examination, positron emission tomography scan, computerized tomography, bone scintigraphy, chest X-ray, and biopsy.^20^ However, due to some limitations of these approaches, such as high cost, time consumption, and age restriction, the development of highly sensitive and early-stage diagnostic techniques required. Different biomarkers such as proteomic biomarkers, gene biomarkers, and various imaging techniques are a useful analytical tool for fast and economic early-stage breast cancer diagnosis.^21^

Breast cancer conventional treatment approach involves; (i) surgical removal of cancer cells. (ii) use of chemotherapy coupled with hormonal therapy and gene therapy; and (iii) radiation therapy.^22^ Surgery is considered as the earliest method and used for most of the solid tumors.^22^ The surgical treatment depends on the stage and tumor form; involves removal of the only lump (lumpectomy) or surgical removal of the entire breast (mastectomy). Breast-conserving surgery includes lumpectomy (removal of lump only or a small number of surrounding tissues), wide excision (partial mastectomy), and quadrantectomy (removal of about one-quarter of the breast.^12^ Currently, sentinel lymph node dissection has become a well-known suitable technique as it necessitates the excision of very few lymph nodes, causing very few or no side effects. Over the past decade, advances in sentinel lymph node mapping have enhanced the precision of detecting sentinel lymph nodes from 80% to 92%-98% using different combined modalities.^12,22^

Chemotherapy is the most conventional therapy available for malignant cancers.^22^ In chemotherapy, anticancer drugs, orally or intravenously given to patients, might cause severe side effects due to non-specific killing of cancer cells. However, radiotherapy is a conventional approach used in the treatment of cancer, along with chemotherapy.^22^ For the treatment of HER2-neu positive tumors, trastuzumab, in combination with radiotherapy, is needed.^23^ Hormonal therapy studied for the treatment of ER+ breast cancer for several decades. The anticancer drug tamoxifen behaves as an antagonist in the breast, causing a delay in thetranscription of estrogen-regulated genes and interrupting in the proliferative effects of estrogen in the breast. Similarly, fulvestrant acts as tamoxifen, but it causes degradation of the ER protein and loss of estrogen and progesterone receptor expression.^24^ Menopausal hormone therapy usually restrained from breast cancer survivors because of the risk of reoccurrence. Menopausal hormone therapy provides adequate assistance from climacteric symptoms, but few are associated with enhanced risk of stroke and also breast, ovarian, and endometrial cancers.^25^ Gene therapies have developed as promising new treatments for breast cancer. Proto-oncogene and tumor suppressor genes have shown accelerated improvement in gene therapy approaches.^12^ Various clinical trials are ongoing to deliver p53 to cancer cells. The viral vectors have employed to transfer a breast cancer gene BRCA1, as a mutation in BRCA genes is also responsible for breast cancer cases. Also, the use of antisense strategies in clinical trials considered the most common approach. Adenoviral gene E1A that interferes with the transcription of erbB-2 can use to inhibit the transcription of overexpressed oncogenes in the treatment of ovarian and breast cancer.^12^

## Plant-derived anti-breast cancer therapeutic agents

**Figure 1:**
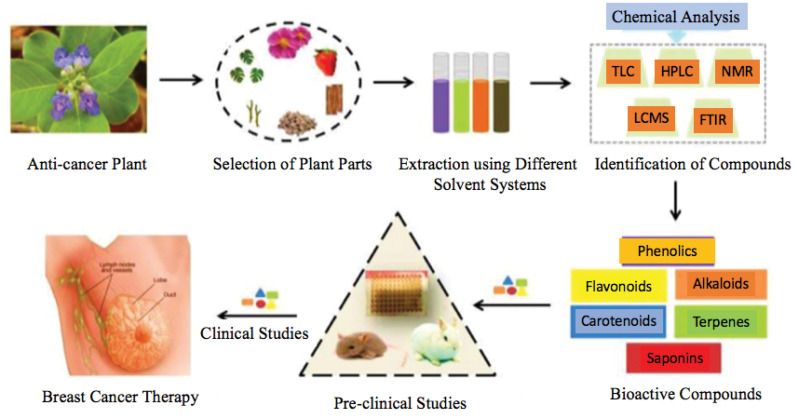
Schematic illustration of plant-derived anti-breast cancer therapeutic agent

Natural products played a remarkable role in the prevention and treatment of cancer and remained a focus of research in drug discovery.^26^ Over 3000 plant species reported having anticancer properties.^27^ Plant-derived natural products have significant efficacy in cancer treatment due to reduced adverse side effects as compared to conventional chemotherapy.^28^ This review article’s primary objective was to study the therapeutic potential of natural products in whole plant extracts/fractions or isolated secondary metabolites in breast cancer treatment. The initial stage in drug discovery is to screen the potential extracts and fractions, which gives the clue of the presence of novel phytochemicals. The knowledge provides a better understanding of the presence of various phytochemicals and their synergistic approach, which nowadays played a remarkable role in combination drug therapy. The extracts/fractions screening reduces the cost of isolation of phytochemicals by revealing a potential agent’s presence. The isolated secondary metabolites from various natural sources, mechanistic study (in-vitro), give better molecular fundamental knowledge of the future therapeutic agent. Figure 1 describes the preparation of phytochemical compounds isolated from plants and their use in breast cancer therapy.

## Anti-breast cancer therapeutic agents and their molecular mechanistic targets

**Figure 2:**
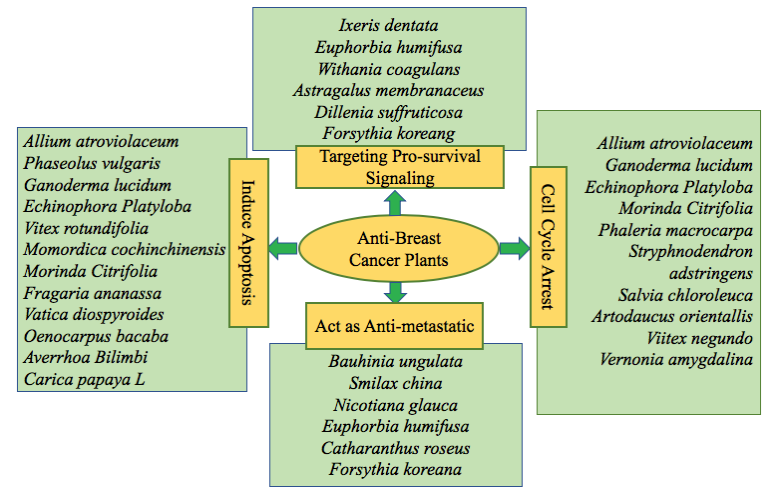
Anti-breast cancer plants and their therapeutic approaches

Several crude extracts/fractions possessing potential natural products have tested on a variety of breast cancer cell lines. The potential phytochemicals induced cytotoxicity on breast cancer through several mechanisms. Such as via induction of apoptosis, cell cycle arrest in cancer cells, inhibition of metastatic potential, obstructing the process of angiogenesis, pro-survival signaling, and autophagy activation. Figures 2 and 2 provide detailed information about potential therapeutic approaches of several anti-breast cancer plant species and their mechanism of action, respectively.

**Figure 3:**
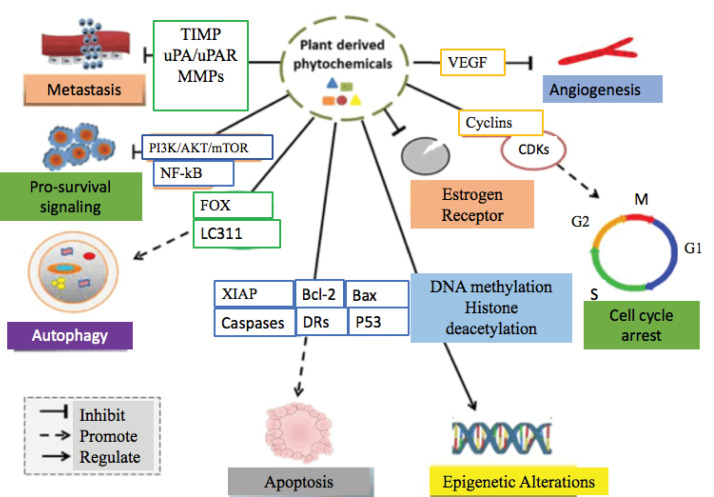
Therapeutic targets of plant derived anti-breast cancer agents on breast cancer cells

### Induction of apoptosis

Apoptosis, tightly regulated mechanism of cell death as a result of signal cascades involved during healthy development and morphogenesis.^3,29^ The enzymatic proteins caspases are prominent initiators and executioners in the process of apoptosis. Along with caspases, various pro and anti-apoptotic proteins such as Bcl-2 family proteins, tumor suppressor proteins (p53), cytochrome c release from mitochondria, activation of several death receptors involved in the trigger of apoptosis. Besides, various apoptosis proteins (IAPs) play a vital role in the induction and regulation of apoptosis.^30,31^ Apoptosis can occur via both the extrinsic pathway (death receptor-mediated pathway) and the intrinsic pathway (mitochondrial-mediated), and these pathways converge at the execution pathway of apoptosis.^3^ Death receptors, DR4, DR5 trigger the extrinsic pathway of apoptosis, up-regulation of pro-apoptotic protein (Bax) and down-regulation of an anti-apoptotic member of Bcl-2, which is essential for the activation of the intrinsic pathway of apoptosis.^3,32^ Extract of *Phaseolus vulgaris* (family Fabaceae) induces apoptosis in MCF-7 and MDA-MB-231 via up-regulating pro-apoptotic protein (Bax) and down-regulated anti-apoptotic protein (Bcl-2, Bcl-xL).^33^ Similarly, fruit extract of *Momordica cochinchinensis* causes the induction of apoptosis in breast cancer (MCF-7) cells via the up-regulation of Bax and enhanced caspase 6, 8, and 9 activity.^34^ Also, *Fragaria ananassa* (Strawberry) methanolic extract induced apoptosis by an intrinsic pathway in T-47D by the up-regulation of Bax, Bid, p73, and down-regulation of BCL-xL.^35^ The aqueous extract fraction of *Oenocarpus bacaba* also induced apoptosis in MCF-7 cells by both extrinsic and intrinsic pathways through activation of caspases-6, -8, and -9.^36^ Moreover, the methanolic fractions of *Scrophularia oxysepala* cause caspase-dependent apoptosis in MCF-7 cells.^37^ The up-regulation of Bax induces apoptosis in MDA-MB-468 cells, treated with acetone and methanolic extracts of *Vatica diospyroides*.^38^

### Modulating cell cycle arrest

Cell cycle, remarkable role in cellular genomic integrity, and timely progression of cells.^39^ Different phases such as (i) G1-phase (gap 1), (ii) S-phase (DNA synthesis), (iii) G2-phase (gap 2), and (iv) M-phase (mitosis) involve in the cell cycle. In S-phase, DNA synthesis and genome replication occur, required for the transmission of genetic information between generations. The M-phase causes segregation of genetic information, sister chromatids, and cell division. G1 is the gap between M and S phase, while G2 is the gap between S and M phase. These intervals (G1 and G2), essential to ensure that each phase is complete before moving to the next phase.^40,41^ Activation of cell cycle check-points usually occurs as a response to replication stress and DNA damage. The activation and inactivation of cyclin-dependent kinases and cyclins play a vital role in the cellular progression and cell cycle regulation.^39,41^ The methanolic extract prepared from *Allium atroviolaceum* (family Amaryllidaceae) induces apoptosis by modulating cell cycle arrest in caspase-dependent and p53-independent pathway in the breast cancer cell (MCF-7, MDA-MB-231).^42^ Similarly, ethanol extract of *Ganoderma lucidum* chipped fruiting bodies causes cell cycle arrest in MCF-7 cells by up‐regulation of p21/Waf1 and down‐regulation of cyclin D1.^43^ The crude extracts of *Echinophora platyloba*, *Vernonia amygdalina*, *Morinda Citrifolia* induces apoptosis in MCF-7 and MDA-MB-231 cell lines via G0/G1/S phase cell cycle arrest.^12,44,45^ The diethyl-ether extract of *Artocarpus altilis* and hexane and methylene chloride fractions from roots of *Salvia chloroleuca* induced apoptosis and sub-G1 peak in T-47D and MCF-7 cells respectively.^46,47^ Also, ethyl acetate fraction from *Phaleria macrocarpa* (fruit) induce G0/G1 and G2/M cell cycle arrest in MDA-MB-231.^48^

### Inhibition of invasion and metastasis suppression

The conventional therapeutic approaches quite challenging, especially in metastasized cancer. The mechanism of metastasis involves invasion, intravasation, and extravasation. The process of invasion characterizes by the spreading of cancer cells to distant sites via the circulatory system. However, extravasation requires the penetration of cancer cells to the endothelium and the basement membrane. At the point of extravasation, cancer cells can grow at secondary focus.^49,50^ The matrix metalloproteinases (MMPs), critical proteins involved in metastasis of tumor cells. The inhibition or blocking of MMPs is an essential target in the suppression of metastatic potential. Other than MMPs, metastasis suppressor genes, MKK4 (mitogen-activated protein kinase 4), BRMS1 (breast cancer metastasis suppressor 1) and NM23 (non-metastatic gene 23) also play a remarkable role in the inhibition of metastasis.^50,51^ Similarly, modulation of uPA, uPAR, and TIMP expression also plays a vital role in the suppression of metastasis.^52^ The crude extracts of *Catharanthus roseus*, *Origanum majorana*, and *Brassica oleracea* possess anti-invasive and anti-metastatic activities in breast cancer cell line, MDA-MB-231. Anti-invasive and anti-metastatic activities via suppression of MMPs (MMP-2 and MMP-9) activities.^53-55^ Similarly, ethanol extract of *Smilax china* causes suppression of metastasis via modulation of uPA, uPAR, and TIMP expression in MDA MB 231 cells.^56^ Also, different fractions from stem of *Bauhinia ungulata* anti-metastatic decrease the activity of potential target of metastasis MMP-2.^57^

### Pro-survival signaling pathway

Several pro-survival signaling pathways were determining the fate of a cancer cell and mainly transduced by a complex net of signaling molecule cascade. Pro-survival signaling cascades, IP3K-PKB/Akt, and MAPK, activated by several cytokines and growth factors. The nuclear factor-κB (NFκB) plays an essential role in the regulation of inflammation and immune responses.^58^ Blocking of these pro-survival signaling pathways has been widely studied, crucial for the treatment of breast cancer. The previous study shows that methanol extract of *Ixeris dentata* induced apoptosis in T-47D, MCF-7, SK-BR-3, and MDA-MB-231 via inhibiting Akt and NF-κB signaling pathway.^59^ Similarly, ethyl acetate fraction of *Euphorbia humifusa* causes inhibition of NF-κB activity in MDA-MB-231 cell line.^60^ Water- ethanol extract of *Astragalus membranaceus* induced apoptosis in MCF-7, SK-BR-3, and MDA-MB-231 through inhibition of PI3K, Akt and mTOR signaling pathways.^61^ Also, ethyl acetate extract from Roots of *Dillenia suffruticosa* induces apoptosis in MCF-7 via inhibition of AKT and ERK, and activation of JNK.^62^

### Other potential pathways

Various signal cascades induce cytotoxicity of breast cancer cell lines via regulation of angiogenesis, autophagy, suppression of ERα expression, down-regulation of intracellular ROS generation, and mitochondrial membrane potential activated. The ethanol crude extract of *Salvia triloba* possesses angiogenesis activities in MCF-7 that is mediated by the inhibition of VEGF expression at both mRNA and protein levels.^63^ Similarly, ethyl acetate fractions of *Eugenia jambolana* and *Musa paradisiaca* causes suppression of VEGF-induced angiogenesis in MCF-7 and MDA-MB-231 cells.^64^ The extract of *Buxus sempervirens* induces autophagic cell death in MCF7, T47D, MCF10CA1a, and BT-20.^65^ However, ROS mediated apoptosis in MCF-7 and MDA-MB-231 noticeable after treatment with chloroform fraction of *Tinospora cordifolia*.^66^ Similarly, the hexane and methylene chloride fractions of *Salvia chloroleuca* also induce ROS-mediated pathway in MCF-7 cells.^46^ Also, *Morinda citrifolia* (ethyl-acetate) extract downregulates intracellular ROS generation and mitochondrial membrane potential in MCF-7, and MDA-MB-231.^45^ The *Acanthopanax sessiliflorus* (hexane fraction) causes mitochondria associated with both ROS dependent and independent pathways in MDA-MB-231 and MCF-7.^67^

## Phytochemicals in anti-breast cancer drug development

Plants possess different phytochemical compounds, and they classified based on the functional group, structures, and biosynthetic origins. Phytochemicals in medicinal plants include phenolics, flavonoids, alkaloids, terpenoids, carotenoids, saponins, steroids, and antioxidants induces cell death in MCF-7 cell lines.^68-86^ Among all phytochemicals, phenolics are the most structurally diverse.^86^ Here, we discuss potential phytochemicals as future anti-breast cancer therapeutic agents and drug development.

### Phenolics

Phenolic compounds, widely occurring secondary metabolites isolated from plants and are most structurally diverse among all phytochemicals.^87^ Plant-derived phenolic compounds classified as; (i) simple phenols, (ii) flavonoids, (iii) lignins, (iv) lignans, (v) tannins, (vi) xanthones, and (vii) coumarins. Previous studies show that various phenolic compounds inhibit the initiation and progression of a variety of cancers by inducing cell cycle arrest, angiogenesis and apoptosis, modulating ROS levels and inhibiting oncogenic signaling cascades controlling cell proliferation.^88^ The quercetin induces apoptosis in MCF-7, T47D, MDA-MB-453 and MDA-MB-231 cell line by up-regulation of Bax, down-regulation of Bcl-2 and activation of caspase-3. Similarly, quercetin also results in cell cycle arrest via modulation of Foxo3a activity in breast cancer.^89-94^ Interestingly, luteolin causes cytotoxicity in breast cancer cell line, MDA-MB-231 via suppression of epidermal growth factor receptor-mediated pathway IGF-1 pathway-dependent ERα.^95^ Moreover, different phenolic acids such as ferulic acid, caffeic acid, and gallic acid also induces apoptosis in ER^+^ and ER^–^ breast cancer cell lines.^88,96,97^

### Alkaloids

Plant-derived alkaloids possess oncogenesis suppression via modulating critical signaling pathways in human cancer. The paclitaxel possesses anticancer activity against breast cancer, ovarian cancer, prostate cancer, and lung cancer and is in clinical use.^98^ Similarly, vinca alkaloids clinically used to treat human cancers. The vinca alkaloids (VA), from the *Madagascar periwinkle* plant (*Catharanthus roseus* G. Don), possess hypoglycaemic and cytotoxic properties. The VA considered as cancer fighters, second-most-certified class of cancer drugs. The four major vinca alkaloids include; (i) vincristine, (ii) vinblastine, (iii) vinorelbine, and (iv) vindesine are in clinical use. The vinflunine, a new synthetic vinca alkaloid, is used in treatment for carcinomas and other malignancies.^99^ The vinca alkaloids interaction with tubulin protein, interfere with the assembly of microtubules, leads to cell division arrest in metaphase.

Similarly, vinflunine, a potent inhibitor of tubulin, causes hindrance in microtubule assembly and induces apoptosis. Moreover, vinflunine apoptosis mechanism involved activation of caspases 3 and 7 and c-Jun N-terminal kinase 1.^100^ Other than vinca alkaloids; another alkaloid compound berberine induces cell cycle arrest and mitochondrial or intrinsic pathway in MCF-7 and MDA-MB-231 cells.^101,102^ Similarly, noscapine induces apoptosis in breast cancer cells via intrinsic and extrinsic pathways by upregulation of Bax, downregulation of Bcl-2 and activation of caspases.^103-105^ The hirsutine causes cell death in MDA-MB-231 cells by activating the intrinsic pathway of apoptosis and targeting NF-κB signaling pathway.^106,107^ Moreover, the treatment of MCF-7 cells by procaine decreases DNA methylation and RARβ2 promoter methylation.^108^

### Terpenes

Terpenes or terpenoids classified based on the number of C5 units or cyclic structures present in the molecule.^109^ The terpenoids can exert a broad spectrum of biological activities such as antioxidation, anti-inflammation, and anticancer activities. Numerous terpenoid compounds are known to possess anticancer potential in a verity of human cancers by causing inhibition of cancer cell proliferation and inducing apoptosis. Monoterpenoids such as D-limonene, have demonstrated antitumor and anticancer activities against breast cancer.^110^ Several diterpenoids also possess anticancer activity against breast cancers and are involved in the induction of apoptosis. These include triptolide, oridonin, and ponicidin.^111^ The triptolide also possesses antiproliferative activity and down-regulates the expression of ERα in different breast cancer cell lines.^112-114^ The triterpenoids are close to steroids in structure and evoke apoptosis in a variety of cancer such as prostate and breast cancer. Different triterpenoids, like cucurbitacins, dammaranes, friedelanes, limonoids, lanostanes, lupanes, oleananes, tirucallanes, and ursanes, have been isolated from plants and studied for anticancer efficacy in breast cancer cells.^115^ Ursolic acid, a triterpene acid causes DNA fragmentation induced apoptosis in MCF-7 cells by downregulation of Bcl-2 and activation of caspase -3.^116,117^ Tetraterpenes also was known as carotenoids broadly categorized as acyclic tetraterpenoids and bicyclic tetraterpenoids. Carotenoids or tetraterpenoids such as lycopene and lutein are also known to possess anticancer activities in breast cancer cell lines.^111^

### Saponins

Saponins are natural glycosides widely distributed in plants classified into; triterpenoid, saponins, and steroid saponins.^118^ Saponins possess potential biological activities includes; anti-inflammatory, antiproliferative, immunomodulatory, and anticancer activities.^119^ Several saponins possess anticancer activities against various cancer cell lines.^120^ For example, Avicin D, a triterpenoid glycoside compound, induces apoptosis in cutaneous T-cell lymphoma cells via downregulation of p-STAT-3 and bcl-2.^121^ Similarly, tubeimoside-1 exhibits anticancer effects via mitochondrial dysfunction and endoplasmic reticulum stress pathways in HeLa cells.^122^ The steroid saponins, degalactotigonin, and Polyphyllin D, possess cytotoxicity activity in ER+ human breast cancer cell line, MCF-7.^123,124^ Moreover, the triterpene saponins such as gummiferaoside B and C possess antiproliferative activity in MDA-MB-435 cells.^125^ Also, Avicins D, G induce apoptosis and cell cycle arrest in MDA-MB-435 cell line.^126^ The phenolics, alkaloids, terpenoids and saponins derived from other sources also possess anticancer activity in different breast cancer cell lines.^127-142^ Moreover, plant extracts, phytochemicals, and their potential mechanism of action against breast cancer are enlisted in Tables 1 and 2, respectively. Also, our various studies show the induction of cell death (apoptosis) in other cell lines.^143^ The induction of cell death mainly due to presence of potential phytochemicals such as phenolics, saponins, terpenoids. The more screening and mechanistic studies need to be done to fully explore the potent phytochemicals in field of cancer therapeutics.

**Table 1:** Plant extracts and their potential mechanism of action against breast cancer

**Plant name**	**Extract / Fraction**	**Part used**	**Target cell lines**	**Mechanism of cell death**	**References**
*Allium atroviolaceum*	Methanolic Extract	Flower	MCF-7, MDA-MB-231	- Induces apoptosis - Modulating cell cycle arrest - Caspase-dependent and p53-independent Pathway	^42^
*Phaseolus vulgaris* (black turtle bean)	Extract	Seeds	MCF-7 and MDA-MB231	- Upregulation of Bax and downregulation of Bcl-2 and Bcl-xL - Activation of caspase -3/7	^33^
*Ganoderma lucidum*	Ethanol extract	Chipped fruiting bodies	MCF-7	- Induces cell cycle arrest and apoptosis - Up‐regulation of p21/Waf1 and down‐regulation of cyclin D1 - Up‐regulation of pro‐apoptotic Bax protein	^43^
*Echinophora Platyloba*	Methanol Extract	Leaves	MDA-MB-231	- Induces apoptosis and cell cycle arrest at S-phase - Up-regulation of bax and p27 - Down-regulation of bcl-2	^44^
*Momordica cochinchinensis*	Aril Extract	Fruit	MCF-7	- Induces apoptosis - Increased bax enhanced caspase 6, 8 and 9 activity	^34^
*Morinda Citrifolia*	Ethyl-acetate extract	Fruit	MCF-7, MDA-MB-231	- Arrested the cell cycle in the G1/S phase in MCF-7 and G0/G1 phase in MDA-MB-231 cells - Downregulation of intracellular ROS generation and mitochondrial membrane potential	^45^
*Fragaria ananassa* *Strawberry*	Methanolic extract	Fruit	T-47D	- Cleavage of MCL-1 - downregulation of BCL-xL - Upregulation of expression of proapoptotic proteins such as BAX and BID - Upregulation of p73 - Activation of CASPASE 3 and CASPASE 9	^35^
*Vatica diospyroides*	Acetone and methanolic extracts	Fruit	MDA-MB-468	- Induces apoptosis - Up-regulation of Bax	^38^
*Oenocarpus bacaba*	Phenolic extract	Fruit	MCF-7	- Induces apoptosis - Caspases-6, -8 and -9 activated	^36^
*Averrhoa Bilimbi*	Methanolic extract	Fruit, Leaves	MCF-7	- Anticancer activity	^68^
*Carica papaya* L	Aqueous Extract	Leaves	MCF-7	- Anti-proliferation and Apoptosis induction	^69^
*Mimosa caesalpiniifolia*	Ethanolic extract	Leaf	MCF-7	- Induces apoptosis - DNA fragmentation	^70^
*Annona muricata*	Aqueous extract	Leaves	MCF-7, MDA-MB-231	- Induces apoptosis	^71^
*Acanthopanax sessiliflorus*	Hexane fraction	Stem bark	MDA-MB-231 and MCF-7	- Non-apoptotic cell death via mitochondria associated with both ROS dependent and independent pathways	^67^
*Phaleria macrocarpa*	Ethyl acetate fraction	Fruit	MDA-MB-231	- Induce G0/G1 and G2/M cell cycle arrest - Activation of caspase -8,9 and 3 - Upregulation of Bax, Bid - cytochrome c, p21, p27, p53 and SMAC - Downregulation of Bcl-2, Bcl-w, XIAP and survivin	^48^
*Stryphnodendron adstringen*	Aqueous extract fraction	Leaves	MCF-7, MDA-MB-435	- Upregulation of Bax, caspase-9, active caspase-3, - caspase-8, LC-3, and beclin-1 - Downregulation of Bcl-2	^72^
*Avicennia Marina*	Crude methanol extract and fraction	Leaves	MDA-MB 231	- DNA fragmentation - Decreased mRNA expression level of Bcl-2 and increased p53	^73^
*Salvia chloroleuca*	Hexane and methylene chloride fractions	Roots	MCF-7	- Induced a sub-G1 peak - DNA fragmentation - ROS-mediated pathway	^46^
*Scrophularia oxysepala*	Methanolic subfractions	Aerial parts	MCF-7	- Activation of caspase-3 - Downregulation of Bcl-2	^36^
*Artocarpus altilis*	Diethyl ether extract	Wood	T-47D	- Induced apoptosis and sub-G1 phase formation	^47^
*Piper crocatum*	Methanol extract	Leaves	T-47D	- Inhibition of p44/p42 phosphorylation	^74^
*Pistacia atlantica*sub*kurdica*	Methanol	Fruits skin	T-47D	- Activation of caspase 3 - Poly ADP ribose polymerase (PARP) cleavage	^75^
*Vitex rotundifolia*	fraction	leave	MCF-7	- extrinsic and intrinsic pathway	^76^
*Vitex rotundifolia*	fraction	leave	T47D	- extrinsic and intrinsic pathway	^77^
*Aaptos sp., marine*	fraction	whole	MCF-7	- DNA fragmentation	^78^
*Marine sponges*	Methanol extract	whole	MCF-7	- DNA fragmentation	^79^
*Vitex negundo*	Aqueous and Ethanolic extract	Leaves	MCF-7	- Induced apoptosis	^80^
*Jatropha curcas*	Ethanol extract	Root bark	MCF-7	- Inducing anoikis	^81^
*Vernonia amygdalina*	Ethanol extract	Leaves	MCF-7 and MDA-MB-231	- Induced apoptosis - G1/S phase cell cycle arrest - Caspase-dependent	^45^
*Strobilanthes crispa*	Hexane extract	Stem	MDA-MB-231	- Induced apoptosis	^82^
*Ixeris dentata*	Methanol extract	***-***	T-47D, MCF-7, SK-BR-3, and MDA-MB-231	- Induced apoptosis - via Akt-NF-κB signaling	^59^
*Tinospora cordifolia*	Chloroform fraction	Stems	MCF-7 and MDA-MB-231	- ROS mediated apoptosis	^66^
*Smilax china*	Ethanol extract	Bark	MDA-MB-231	- Suppression of metastasis Modulation of uPA, uPAR and TIMP expression	^56^
*Bauhinia ungulata*	Different fractions	Stem	4T1	- Anti-tumor - Antimetastatic - decreasing the MMP-2 activity	^57^
*Nicotiana glauca*	Dichloromethane fraction	Stem	MCF-7	- Anti-Metastatic	^83^
*Euphorbia humifusa*	Ethyl acetate fraction	Whole plant	MDA-MB-231	- Inhibition of NF-κB activity - Induced matrix metalloproteinase (MMP)-9 mRNA expression	^60^
*Withania coagulans*	Ethyl acetate	Aerial with fruit	MCF-7, MDA-MB-231	- Inhibited TNF-α induced NFκB activity	^84^
*Astragalus membranaceus*	Water- ethanol extract	Roots	MCF-7, SK-BR-3 and MDA-MB-231	- Anti-proliferative - Induced apoptosis - Inhibition of PI3K/AKT/mTOR signaling pathway	^61^
*Dillenia suffruticosa*	Ethyl acetate extract	Roots	MCF-7	- Induces apoptosis via inhibition of AKT and ERK, and activation of JNK	^62^
*Catharanthus roseus*	Methanol extract	Leaves	MDA-MB-231	- Anti-invasive - Suppressed the MMP-2 and MMP-9 activity	^53^
*Forsythia koreana*	Methanol extract	Fruit and leaves	MDA-MB-231	- Suppressed invasion and MMPs activities - Inhibited the receptor activator of nuclear factor kappa-B	^85^
*Origanum majorana*	Ethanolic extract	Leaves	MDA-MB-231	- Anti-invasive and anti-metastatic - Downregulates the phosphorylation of IκB, nuclear level of NFκB and Nitric Oxide (NO) production	^54^
*Brassica oleracea*	Extract	-	MDA-MB-231	- Anti-invasive - Suppressed TPA-induced MMP-9 activity	^55^
*Salvia triloba*	Ethanolic crude extracts	Whole plant	MCF 7	- Antiangiogenesis - Inhibited the expression of VEGF at the mRNA and protein level	^63^
*Eugenia jambolana*	Ethyl acetate fractions	Seeds	MCF-7 and MDA-MB-231	- Suppression of VEGF-induced angiogenesis	^64^
*Musa paradisiaca*	Ethyl acetate fractions	Roots	MCF-7 and MDA-MB-231	- Suppression of VEGF-induced angiogenesis	^64^
*Buxus sempervirens*	Acetonic extract	Leaves and flowers	MCF7, T47D, MCF10CA1a and BT-20	- Induces apoptosis, Cell cycle arrest, autophagy	^65^

**Table 2:** Plant derived phytochemicals and their potential mechanism of action against breast cancer

**Phytochemicals**	**Compound name, type**	**Target cell lines**	**Mechanism of cell death**	**References**
Phenolics	Quercetin (Flavonoid)	MCF-7, T47D, MDA-MB-453, MDA-MB-231	- Induces apoptosis - Through suppression of Twist via p38 MAPK pathway - Increased Bax expression and decreased Bcl-2 expression - Increased cleaved caspase-3 and PARP expression - Cell cycle arrest through modulation of Foxo3a activity	^89-94^
	Casticin (flavonoid)	MCF-7, MDA-MB-231	- Induces apoptosis - Inhibiting the expression of forkhead box protein M1	^127^
	Luteolin (flavonoid)	MDA-MB-231, MCF-7	- Suppression of epidermal growth factor receptor-mediated pathway - IGF-1 pathway dependent ERα	^95,128^
	Ferulic acid (phenolic acid)	MDA-MB-231, T47D, MCF-7	- Induces apoptosis - Suppression of metastatic potential - Anti-proliferative	^96,97,129^
	Caffeic acid (phenolic acid)	T47D, MCF-7	- Anti-proliferative, - Induces apoptosis - Inhibition of NFκB and activation of Fas	^97,130^
	Gallic acid (Phenolic acid)	MDA-MB-231	- Induces apoptosis	^88^
Alkaloids	Berberine	MCF-7, MDA-MB-231	- Inducing cell cycle arrest - Increasing levels of cytoplasmic cytochrome c, caspase-9, p53 and p27 - Cleavage of PARP - Decreasing levels of Bcl-2	^101,102,131^
	Noscapine	MCF-7, MDA-MB-231, T47D	- Activation of caspase-8 and caspase-9 - Upregulation of Bax, downregulation of Bcl-2 - Anti-neoplastic	^103-105^
	Pretazettine	MCF-7	- Anti-tumor activity	^132^
	Piperlongumine	MDA-MB-453, MCF-7, T-47D	- STAT3 Inhibitor	^133^
	Hirsutine	MDA-MB-453, MDA-MB-231, 4T1	- DNA damage response - NF-κB and Akt pathways - Activation of the p38 MAPK pathway - Upregulation of Bax, downregulation of Bcl-2 - Activating caspase 9 and caspase 3	^106-107,134^
		Procaine	MCF-7	- Decrease global DNA methylation - Decrease RARβ2 promoter methylation	^108^
		Benzyl Isothiocyanate	MDA-MB-231, MCF-7, MDA-MB-468, BT-474, and BRI-JM04	- FoxO1-mediated autophagic cell death	^135^
Terpenoid	D-Limonene (Monoterpene)			
	Triptolide (Diterpene)	MDA-MB-435, MDA-MB-231, MCF7	- Anti-proliferative - Suppression of phospholipase D expression - Down-regulate the expression of ERα	^112-114^
	Ursolic acid (triterpene acid)	MCF-7	- Triggers apoptosis - DNA fragmentation - Downregulation of Bcl-2 - Activation of caspase -3	^116-117^
	Betulinic acid (triterpene)	MDAMB- 231, MDL13E, BT438, BT474, BT549, T47D	- Exhibited cytotoxicity - Induces apoptosis - Down-regulation of Bcl-2 and cyclin D1	^136-137^
	Lupeol (triterpene)	MDA-MB-231	- Suppressed the proliferation	^138^
	Lycopene (tetraterpenoids, carotenoids)	MCF 7, MDA-MB-231	- Trigger G2/M arrest and suppress Bcl-2 expression - Induce apoptosis	^139^
		Parthenolide	ZR-75-1, MDA-MB-231	- Inhibits HDAC1 increases global H3 acetylation, - Induces p21	^140-141^
Saponins	Gummiferaoside B, C (triterpene saponins)	MDA-MB-435	- Anti-proliferative	^125^
	Degalactotigonin (steroid saponins)	MCF-7	- Cytotoxic	^123^
	Polyphyllin D (Steroid saponins)	MCF-7	- Cytotoxic	^124^
	Avicins D, G (triterpenoid saponins)	MDA-MB-435	- Apoptosis, - Cell cycle (G1) arrest	^126^
	Ginsenoside Rh_2_ (dammarane-type saponins)	MCF7	- Cytotoxic	^142^

## Conclusion

Cancer is a complex disease, leading cause of death worldwide. Despite the development of many synthetic anticancer drugs, toxicity remains the main problem, which reduces the survival rate. Therefore, there is an increase in demand for alternative treatments. Amongst the alternative approaches, the natural product derived anticancer agents are a practical choice. The secondary metabolites, as potential anticancer agents with understandable anticancer mechanisms of action, leads to the development of novel therapeutic drugs. Additionally, the plant extracts are an excellent source of lead compounds. The isolated lead compound can either used or undergoes some structural modifications to increase the effectiveness in term of their pharmacological potential.

## Ethical Issues

Not applicable.

## Conflict of Interest

The authors have no conflict of interest.
